# Type D personality in the general population: a systematic review of health status, mechanisms of disease, and work-related problems

**DOI:** 10.1186/1477-7525-8-9

**Published:** 2010-01-23

**Authors:** Floortje Mols, Johan Denollet

**Affiliations:** 1 CoRPS - Center of Research on Psychology in Somatic Diseases, Department of Medical Psychology, Tilburg University, The Netherlands

## Abstract

**Background:**

The objective was to review all available literature concerning Type D (distressed) personality among the general population and to discuss its implications for research on health status, disease-promoting mechanisms and work-related problems in non-clinical populations.

**Methods:**

A computerized search of the literature was performed independently and in duplicate by both investigators on December 21^st^, 2009. Published research reports were included if they studied Type D personality among the general population. Nineteen articles were selected and they were subjected to an 11-item standardised quality checklist by both investigators.

**Results:**

The methodological quality of the selected studies was adequate to high. The studies included in this review showed that the presence of Type D characteristics had a negative impact on mental health status (more symptoms of depression, anxiety, post-traumatic stress disorder, mental distress, passive coping, and less social support) and physical health status (more somatic complaints, lower health status, more influenza-like illness reporting). Other studies reported on behavioral and biological mechanisms of disease in apparently healthy individuals with a Type D personality. Finally, some studies also showed a negative effect of Type D personality on work-related problems (higher absence-leave, higher levels of vital exhaustion and burnout, and more work-related stress).

**Conclusions:**

Type D personality is a vulnerability factor for general psychological distress that affects mental and physical health status and is associated with disease-promoting mechanisms and work-related problems in apparently healthy individuals.

## Introduction

In the past decade, studies on the effects of Type D personality on clinical and psychological outcomes have been flourishing. Type D personality has been described as the tendency to experience a high joint occurrence of negative affectivity and social inhibition [1]. People that score high on negative affectivity have the tendency to experience negative emotions, while people that score high on social inhibition have the tendency not to express these emotions, because of fear of rejection or disapproval by others. Persons with high levels on *both *personality traits are classified as having a Type D personality [1].

The Type D construct can be measured with the short and easy-to-use DS14 questionnaire [1,2]. It consists of two 7-item subscales assessing negative affectivity (e.g. "I often feel unhappy") and social inhibition (e.g. "I am a closed person") respectively. Individuals are categorized as Type D using a standardized cut-off score ≥ 10 on both the negative affectivity and social inhibition subscales. Correlational studies have shown that Type D personality is different from behavior patterns Type A and Type B [2]. In addition, validation of the Type D construct against the Five Factor Model of personality, showed that negative affectivity correlated positively with neuroticism, social inhibition correlated negatively with extraversion, and both negative affectivity and social inhibition correlated negatively with conscientiousness [1].

The majority of studies on Type D personality have focused on its prevalence and effects in patients with a variety of cardiovascular diseases since the Type D construct was originally described and further developed in this patient group [3]. These studies in cardiovascular patients have shown that Type D personality is an independent predictor of negative health outcomes such as poor health status, (recurrent) myocardial infarction, and increased risk of mortality [4-7]. Given the clinical relevance of findings on Type D research in the context of cardiovascular disorders, it is also important to assess the potential relevance of the Type D construct among apparently healthy people from the general population.

Although Type D personality has been shown to predict cardiac prognosis after adjustment for clinical markers of disease severity [4,7], there still is a possibility that markers of disease severity that were not controlled for might have led to the occurrence of Type D characteristics in these studies. Studying Type D personality in apparently healthy people from the general population would provide a more direct test of the notion that Type D is not an epiphenomenon caused by cardiovascular disorder. Moreover, Type D personality is based on normal personality traits rather than psychopathology which implies that it should be prevalent in the general population as well [1], and that it may have an adverse effect on the perceived health status as reported by individuals from the general population.

Recently, a number of studies have been published on the effect of Type D personality in different subgroups from the general population. The primary aim of the present study was to review all the available evidence concerning Type D personality in relation to mental and physical health status among apparently healthy people from the general population. In addition, we wanted to review the role of Type D personality in potential mechanisms of disease as markers of health risks in apparently healthy people. Finally, we sought to describe potential work-related problems that are associated with Type D personality in economically active populations.

## Methods

### Search strategy

A computerized search of the literature through the search engines Pubmed, Science Direct, and PsychINFO was performed on December 21^st^, 2009, using the terms 'Type D personality' and 'Type D'. Reference lists of all identified publications were checked to retrieve other relevant publications, which were not identified by means of the computerized search.

### Selection criteria

Studies that met the following criteria were included; (1) if the objective was to describe Type D personality in the general population, (2) if the publication was an original article (e.g. no poster abstracts, letters to the editor etc.), (3) if they were published in peer-review journals, and (4) if they were written in English. Studies were excluded for the following reasons; (1) if they included a patient population, and (2) if they only reported results on negative affectivity or social inhibition instead of Type D. The literature search was conducted independently and in duplicate by both investigators.

**Figure 1:**
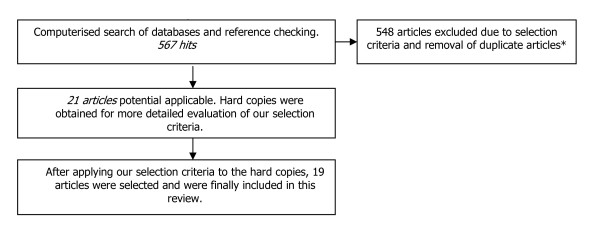
**Flow diagram of papers accepted and rejected during selection procedure**. * The selection criteria are described in the methods section.

The described inclusion and exclusion criteria were applied to our initial 567 hits. Based on their titles and abstracts 21 articles met our criteria. These studies were conducted between 2002 and 2009. Hard copies were obtained of 21 studies and were reviewed by both investigators. After careful review, 19 articles fulfilled our selection criteria and were included in this review [8-26]. A flow-chart of this selection procedure is shown in Figure 1.

### Quality assessment

The methodological quality of each of the selected articles was assessed with an 11-item standardised checklist of predefined criteria by both investigators. The checklist was based on established criteria lists for systematic reviews [27,28]. The criteria are presented in Table 1.

**Table 1:** List of criteria for assessing the methodological quality of studies on the relationship between Type D personality and the general population.

**Positive if with respect to: **	
Type D assessment	
1.	A validated Type D questionnaire is used (e.g. DS16, DS24 or DS14)
2.	The correct method of calculating Type D is used (e.g. as described in the publications associated with the Type D questionnaires)
	
Study population	
3.	A description is included of at least two socio-demographic variables
4.	Inclusion and/or exclusion criteria are described
5.	Participation rates for patient groups are described and are more than 75%
6.	Information is given about the degree of selection of sample (information is given about the ratio respondents versus non-respondents).
	
Study design	
7.	The study size is consisting of at least 50 participants (arbitrarily chosen)
8.	The collection of data is prospectively gathered
9.	The process of data collection is described (e.g. interview or self-report)
	
Results	
10.	The results are compared between two groups or more (e.g., Type D vs. non-Type D, groups with different gender or age etc.)
11.	Statistical proof for the findings is reported

Each item of a selected study, that matched our criteria, received one point. If an item did not meet our criteria or was described insufficiently or not at all, zero points were assigned. The highest possible score was thus 11. Studies scoring 75% or more of the maximum attainable score (= 8 points) were arbitrarily considered to be of 'high quality'. Studies scoring between 50% and 75% (6-7 points) were rated as 'adequate quality'. Studies scoring lower then 50% (i.e. <6 points) were considered to be of 'low quality'.

## Results

### Methodological quality of the studies

The evaluation of the methodological quality of the 19 studies by the two reviewers yielded the following results. On 5 items, there was disagreement between the reviewers, mostly due to differences in interpretation. These were solved through discussion in a consensus meeting. The quality scores ranged from 6 to 9 points (Table 2) and the mean quality score was 8. Thirteen studies were of a high quality although none of the studies received the maximum attainable score [8,11,12,14-16,18,21-26]. The remaining six studies contained adequate levels of evidence [9,10,13,17,19,20]. None of the studies was considered to be of low quality according to our list of quality criteria. General shortcomings were criteria 5 (response rate less then 75%) and criteria 6 (information on the degree of selection of the sample).

**Table 2:** Characteristics of studies*

Study	Country		Size sample	Participants	Mean age in years	Sex	% Type D	Design	Research group	Study quality
[8]	U.K.		564	British males	M = 26.2 R = 18-55	564 men	29%	Cross-sectional	Other	8

[9]	Belgium		155	policemen and nurses	M = 32 R = 20 - 56	66 men 85 women	35.5%	Cross-sectional	C*o*RPS	7

[10]	Netherlands		17	right-handed men	M = 23 SD = 2.4	17 men no women	23.5%	Cross-sectional	C*o*RPS	6

[11]	Canada		173	university students	M = 20.4	86 men 87 women	Unknown	Prospective	Other	9

[12]	Germany		492	employees at manufactory	M = 40.5 SD = 11.4	438 men 54 women	Unknown	Cross-sectional	Other	9

[13]	Netherlands		668	children	M = 10.3 R = 8.6 - 12.8	368 boys 300 girls	27.5%	Prospective	Other	7

[14]	Netherlands		151	prison workers	M = 44.0 R = 22 - 59	111 men 40 women	16.6%	Cross-sectional	Other	8

[15]	Netherlands		3331	healthy twins	M = 17.2 R = 12 - 24	1519 men 1812 women	26.7%	Cross-sectional	C*o*RPS	8

[16]	Netherlands		755	student teachers	M = 18.8 R = 16-29	No men 755 women	25.9%	Cross-sectional	Other	9

[17]	Poland		79	psychiatrists and nurses	M = 39.7 S = 8.2	25 men 28 women	27.8%	Cross-sectional	Other	6

[18]	Ukraine		250	university students	M = 20.9 SD = 3.4	113 men 137 women	22.4%	Prospective	C*o*RPS	9

[19]	U.K.		334	university students	M = 19.5 R = 18-41	180 men 154 women	24.9%	Cross-sectional	Other	7

[20]	Germany		634	employees at manufactory	M = 39.9 SD = 10.7	575 men 67 women	Unknown	Cross-sectional	Other	7

[21]	Netherlands		5404	adults	M = 45.8 SD = 15.9	2697 men 2707 women	Unknown	Prospective	C*o*RPS	9

[22]	Belgium/Netherlands		932	female teachers	M = 25 IQR = 19-42	no men 932 women	28.4%	Cross-sectional	Other	8

[23]	Netherlands		622	adults	M = 54.2 SD = 14	318 men 304 women	18.1%	Cross-sectional	C*o*RPS	8

[24]	Belgium		132	adults	M = 33.7 SD = 14.5	70 men 57 women	Unknown	Cross-sectional	Other	9

[25]	U.K.		84	adults	M = 22.0 SD = 6.8	42 men 42 women	Unknown	Prospective	Other	9

[26]	U.K./Ireland		1012	adults	M = 20.5 SD = 4.8	225 men 787 women	38.5%	Cross-sectional	Other	8

* In alphabetical order

R = range; SD = standard deviation; IQR = interquartile range; C*o*RPS = Center of Research on Psychology in Somatic Diseases; Other = other research group not related to C*o*RPS.

### Study characteristics

All studies were published after 2001. The studied populations included policemen, nurses, psychiatrists, prison workers, employees at a manufacturing plant, female teachers, right-handed males, university students, undergraduates, youngsters, twins, and respondents from the general population (Table 2). Because the studies included children, students, and middle-aged adults, the age range of participants differed between the included studies. The lowest mean age was 10.7 [13] and the highest was 54.2 [23]. Most studies included both males and females.

The most frequently used questionnaire to determine Type D personality was the 14-item DS14 scale [8,10,12,14,17-21,23-26]. In the other studies the DS16 [16,22] or DS24 [9,11] scales were used. One study assessed social inhibition with the social avoidance and distress items of the Social Anxiety Scale for Children and used the Children's Depression Inventory to assess negative affect [13]. Another study assessed Type D by a combination of the Amsterdam Biographical Questionnaire, the Spielberger Trait Anxiety Inventory and the Young Adult Self-Report [15]; after the combination of scales method, 20 items were selected and used to determine Type D status.

Whereas the majority of publications on Type D personality among patients with cardiovascular diseases originate from the Center of Research on Psychology in Somatic diseases (C*o*RPS) at Tilburg University, the Netherlands, only 6 studies in the present review originated from C*o*RPS and 13 studies on Type D in the general population were performed by other research groups. The studies were conducted in populations from 8 different countries: Netherlands (n = 8), United Kingdom (n = 4), Belgium (n = 3), Germany (n = 2), Canada, Ireland, Poland, and Ukraine.

The results of the studies included in this review are first described below according to the impact of Type D personality on mental and physical health status (Table 3). Next, evidence is reviewed regarding the role of Type D in the medical (mechanisms of disease in healthy people) and the occupational (work-related problems in economically active populations) context (Table 4). Because some studies included a variety of outcomes, they are included in more than one category.

**Table 3:** Outcomes of studies: Health status

	Outcome	Study	Participants	Conclusion
**(3a)**	**Mental health status**	[23]	622 adults (Netherlands)	Type D individuals experienced more *symptoms of depression *(r = 0.42; p < 0.01) and *anxiety *(r = 0.35; P < 0.01) compared to non-Type D individuals.

		[9]	155 policemen and nurses (Belgium)	Type D individuals experienced more *symptoms of depression *(9.1 vs. 7.7; p < 0.01) and *anxiety *(14.1 vs. 11.1; p < 0.001) compared to non-Type D individuals.

		[18]	250 university students (Ukraine)	Type D individuals experienced more *symptoms of depression *(p < 0.001), *anxiety *(p < 0.001), and *negative affect *(p < 0.001), as well as *less positive affect *(p < 0.001) than non-Type Ds.

		[13]	668 children (Netherlands)	Children with a Type D personality reported more *negative mood states *(10.43 vs. 6.96) and more *non-productive thoughts *(10.15 vs. 5.13) than non-Type D children.

		[17]	79 psychiatrists and nurses (Poland)	Individuals with a Type D personality manifested significantly more *symptoms of mental health disorders *than non-Type D individuals.

		[24]	132 adults (Belgium)	Type D individuals had more *symptoms of mental distress *(*r*s > .38) compared to non-Type D; Type D has a more adverse effect with low levels of authoritarianism (β = .62; p < 0.01).

		[19]	334 university students (U.K.)	Type D's tend to use more *passive and maladaptive avoidance coping strategies *such as resignation and withdrawal. This is associated with higher levels of perceived stress and burnout symptoms.

		[25]	84 adults (U.K.)	In an experimental research setting, Type D individuals exhibited higher feelings of *subjective stress *compared to non-Type D individuals (F(1.83) = 6.43; p < 0.03).

		[26]	1012 adults (U.K. and Ireland)	Type D individuals reported *lower levels of social support *(12.7 vs. 14.7; p < 0.001), and they were more likely to *let things get them down *(p < 0.001) compared to non-Type Ds.

**(3b)**	**Physical health status**	[13]	668 children (Netherlands)	Children with a Type D personality reported more *somatic complaints *(24 vs. 18; p < 0.05) compare to non-Type D children.

		[23]	622 adults (Netherlands)	Individuals with a Type D personality reported a significantly *lower health status *(all *p*s < 0.01) compared to non-Type D individuals.

		[9]	155 policemen and nurses (Belgium)	Individuals with a Type D personality reported a significantly *lower health status *(50.4 vs. 42.5; p < 0.001) compared to non-Type D individuals.

		[21]	5404 adults (Netherlands)	Negative affectivity was related to *more influenza-like illness *reporting (OR = 1.05, p = 0.009); however, social inhibition to *less influenza-like illness *reporting (OR = 0.97; p = 0.011).

		[22]	932 female teachers (Belgium and Netherlands)	Female teachers with a Type D personality were more *bothered by their voice complaints *(10 vs. 7; p < 0.001) than their non-Type D counterparts.

		[16]	755 student teachers (Belgium and Netherlands)	Type D student teachers had a 4× greater risk of a *high Voice Handicap Inventory score *(rating the subjective biopsychosocial consequences caused by voice problems) than the non-Type D group.

**Table 4:** Outcomes of studies: Medical and occupational setting

	Outcome	Study	Participants	Conclusion
**(4a)**	**Medical: mechanisms of disease**	[22]	932 female teachers (Belgium and Netherlands)	Female teachers with a Type D personality were significantly *less likely to get treatment *for their voice complaints than their non-Type D counterparts (25.7% vs. 39.3%; p = 0.016).

		[26]	1012 adults (U.K. and Ireland)	Type D individuals had *fewer regular medical checkups *(p = 0.027), and were *less likely to eat sensibly *(p = 0.033) or to *spend time outdoors *(p < 0.001) compared to non-Type Ds.

		[8]	564 males (U.K.)	*Body dissatisfaction *is more prevalent in Type D's or in men who are sedentary. The interaction between Type D and being sedentary is detrimental because it can influence health risk behaviors

		[25]	84 adults (U.K.)	Men with a Type D personality, but not women, exhibited *higher cardiac output *during experimental stress compared to non-Type D men (F[3,37] = 3.4; p < 0.05).

		[11]	173 university students (Canada)	Socially inhibited men had heightened systolic and diastolic *blood pressure reactivity *(p < 0.05); negative affectivity was related to *dampened heart rate reactivity *in men (p < 0.05).

		[10]	17 men (Netherlands)	The difference in *amygdala activity *in reaction to fearful vs. neutral face/body expressions was present in non-Type Ds (p = 0.004) but was absent in Type D individuals (p = 0.110).

		[15]	3331 healthy twins (Netherlands)	Type D personality was *substantially heritable *(52%); heritability for negative affectivity was 46%, while heritability for social inhibition was 50%.

**(4b)**	**Occupational: work-related problems**	[12]	492 employees at manufactory (Germany)	Employees with a Type D personality were more often *absent from work *than their non-Type D counterparts (β = 0.499; p < 0.01).

		[20]	634 employees at manufactory (Germany)	Employees with a Type D personality were more likely to report *symptoms of vital exhaustion *than non-Type Ds (r = 0.574; p < 0.001)

		[17]	79 psychiatrists and nurses (Poland)	Individuals with a Type D personality perceived their *workplace as more stressful *and had a higher level of *burnout *than non-Type D individuals.

		[14]	151 prison workers (Netherlands)	Type Ds were more at risk for *post-traumatic stress disorder *than non-Type Ds (OR 9.09; 95%CI = 2.1-39.1; p < 0.005); this risk increased when *exposed to inmate aggression*.

### Type D personality and health status

Eight studies included in this review reported that Type D personality was negatively associated with mental health status (Table 3 - section a). Type D personality was associated with more remembered alienation from parents and control by parents while growing up [23]. Furthermore, adults with a Type D personality experienced more symptoms of depression and anxiety compared to non-Type D adults [9,18,23], and they reported significantly more negative affect and less positive affect compared to non-Type D's [18]. In addition, children with a Type D personality reported more negative mood states and more non-productive thoughts than non-Type D children [13]. Moreover, individuals with a Type D personality manifested significantly more symptoms of mental health disorders [17], had more symptoms of mental distress [24], and exhibited higher feelings of subjective stress than non-Type D individuals [25]. Individuals with a Type D personality also tend to use more passive and maladaptive avoidance coping strategies which is associated with higher levels of perceived stress and burnout symptoms [19]. Finally, Type D individuals reported lower levels of social support, and they were more likely to let things get them down compared to non-Type Ds [26].

Six studies reported results on the effect of Type D personality on physical health status (Table 3 - section b). Children with a Type D personality reported more somatic complaints (24 vs. 18; p < 0.05) compared to non-Type D children [13]. Adult men and women with a Type D personality also reported a significantly lower health status compared to non-Type D's [9,23]. Another study reported that negative affectivity was associated with more influenza-like illness reporting while social inhibition was associated with less influenza-like illness reporting [21]. Finally, female teachers with a Type D personality were more bothered by their voice complaints [22] and reported a higher biopsychosocial impact of their voice complaints [16] than their non-Type D counterparts.

Apart from Type D personality and perceived health status, we also reviewed empirical and experimental evidence regarding the role of Type D personality in potential mechanisms of disease as well as work-related problems in apparently healthy individuals from the general population.

### Type D personality and mechanisms of disease

Six studies examined behavioral and biological mechanisms of disease as a function of Type D personality in apparently health individuals (Table 4 - section a). Regarding behavioral mechanisms, two studies showed that Type D personality was associated a decreased likelihood of getting appropriate medical care. Female Type D teachers with recent voice complaints seek out less (para-)medical care and were less likely to have undergone a treatment for their complaints than their non-Type D counterparts [22]. In another study, Type D individuals were less likely to have a regular medical check-up [26]. In the latter study, Type D was also associated with an unhealthy lifestyle; i.e., Type D individuals were less likely to eat sensibly or to spend time outdoors compared to non-Type Ds [26]. Finally, a recent study showed that body dissatisfaction was more prevalent in men with a Type D personality and in men who are sedentary [8]. The interaction between Type D personality and being sedentary is detrimental to health because it can influence health risk behaviors.

Biological mechanisms of disease in Type D research among healthy populations included the cardiovascular system, emotion-processing in the brain, and heritability. Men with a Type D personality, but not women, exhibited higher cardiac output during experimental stress compared to non-Type D men [25]. Another study showed that socially inhibited men had heightened systolic and diastolic blood pressure reactivity, while negative affectivity was related to dampened heart rate reactivity [11]. Type D was also associated with a differential activity of the amygdala in reaction to fearful versus neutral face and body expressions. Emotion-evoked activation of the amygdala was present in non-Type D's but was absent in Type D individuals [10]. Finally, evidence suggests that Type D personality may be substantially heritable; heritability has been estimated to be 52% [15]. Heritability for negative affectivity was 46% due to additive genetic factors, while heritability for social inhibition was 50% due to nonadditive or dominance genetic effects [15].

### Type D personality and work-related problems

Associations between Type D personality and impaired health status may also have an impact on health problems in the occupational setting. Four studies reported that a Type D personality was associated with work-related problems (Table 4 - section b). With reference to this issue, Type D personality has been associated with effort-reward imbalance, overcommitment, perceived adverse physical working conditions, and substantial problems in interactions with supervisors and co-workers [12]. Importantly, this study also showed that employees with a Type D personality were more often absent from work than their non-Type D counterparts [12]. A possible explanation for this higher rate of sick-leave is the fact that employees with a Type D personality are more likely to report symptoms of vital exhaustion [20], and perceive their workplace as more stressful [17]. Employees with a Type D personality also have higher levels of burnout, and show a lower sense of personal accomplishment [17]. Type D employees may be up to 9 times more likely to develop post-traumatic stress disorder than non-Type D's, especially when they are confronted with significant stressors at work [14].

## Conclusions

Although the majority of studies on Type D personality has focussed on cardiovascular [1,3-7,29-34], or other medical populations [35], this systematic review indicates that Type D may negatively affect health status of apparently healthy individuals from the general population as well.

First, the studies included in this review showed that the presence of Type D personality had an adverse effect on mental health status. Various studies showed that individuals from the general population with a Type D personality experienced more symptoms of distress, depression and anxiety compared to non-Type D's [9,13,18,19,23-25]. This increased vulnerability for mental health problems in Type D individuals was also found in chronic pain patients [36], diabetes patients [37], and cardiac patients [38]. Furthermore, the studies included in this review showed that people with a Type D personality more often reported mental health disorders [17] as well as lower levels of social support [26] compared to non-Type D adults.

The presence of Type D personality among people from the general population was also associated with a poor physical health status. For example, Type D's reported more somatic complaints [13,16,22] and a significantly lower health status compared to non-Type D's [9,23]. This is in line with the adverse effects of Type D on somatic health status in cardiovascular conditions. In patients with heart failure, it was found that Type D personality was an independent predictor of impaired health status [39] and more cardiac symptoms [40]. Also, Type D patients with heart failure were at 6-fold increased risk of reporting impaired health status compared to the reference group of non-Type D patients [41]. Finally, Type D was a strong predictor of adverse cardiac outcome after acute myocardial infarction, and the associated risk was similar to that of traditional cardiovascular risk factors [7].

Some studies that are included in this review explored the behavioral and biological mechanisms of disease as a function of Type D personality in apparently health individuals. Hence, a poor physical health status can be explained by the fact that Type D individuals perform significantly fewer health-related behaviors (eat sensibly, spend time outdoors, get a regular medical check-up) [26] and that they are more likely to smoke [6] as compared to non-Type D individuals. Furthermore, two studies showed that individuals with a Type D personality are less likely to seek appropriate medical care [22,26]. This has also been shown in Type D patients with chronic heart failure causing a significant decrease in health status among these patients [41,42].

The fact that Type D individuals tend to experience interpersonal situations as being stressful may also have direct biological effects that may impact on the cardiovascular system. Responding to these situations can elicit physiological reactivity every time a potentially "threatening" situation is encountered [11]. Accordingly, Type D was associated with increased cardiac output [25], heightened systolic and diastolic blood pressure reactivity [11], and dampened heart rate reactivity during experimental stress. Type D was also associated with a decreased activity in the amygdala in response to fearful expressions [10], suggesting inadequate emotion-processing in the brain. Finally, heritability might be an underlying third variable that explains the co-occurrence of disease and Type D personality through a shared genetic component that predispose people to both physical and psychological distress. In fact, Type D personality has been shown to be substantially heritable [15] and research on genetic linkage has provided more evidence for the biological underpinnings of the Type D construct [43].

Clinical research in cardiac populations confirmed that Type D personality was independently associated with indices of cardiovascular reactivity such as reduced heart rate recovery [44]. Other findings from clinical research also pointed towards neuroendocrine and immunological pathways that may explain the adverse health outcomes associated with Type D personality. Type D personality has been associated with elevated levels of the stress hormone cortisol [45], increased oxidative stress [46], immune dysfunction, and decreased numbers of bone-marrow derived endothelial progenitor cells [47] in cardiac patients. These initial findings are promising, but more research is needed to examine the cardiovascular effects of stress in apparently healthy individuals with a Type D personality. Hence, future research should also focus on neuroendocrine and immunological mechanisms that may advance our understanding of biological pathways in non-clinical populations.

The presence of Type D personality may also be associated with health-related problems in the occupational setting. Type Ds were more often absent from work [12], were more likely to report symptoms of vital exhaustion [20] or post-traumatic stress disorder [14], perceived their workplace as more stressful, had higher levels of burnout, and showed a lower sense of personal accomplishment [17] than non-Type D's. To our knowledge, only one other study investigated the relationship between work and Type D personality in patients with an acute coronary syndrom, and found that failure to resume work was not related to Type D personality [48].

This review has some limitations. The cross-sectional nature of most studies (14 out of 19) did not allow us to determine causal associations between Type D and the studied outcomes. A prospective study might provide us with more answers about the exact relationship between Type D personality and specific outcomes and the extent of this relationship. In addition, the studies included in this review used a number of different questionnaires to assess Type D personality. Also, not all studies used the correct method of calculating Type D. Some studies claim to report on the effects of Type D personality on health but only report on the effects of social inhibition and negative affectivity on health. Standardisation of the use of valid Type D questionnaires is essential for adequate evaluation and mutual comparison of studies. Finally, one study reported on the effect of Type D personality in children [13]. Although the results of this study were similar to the results found in studies among adults, we need to be careful with drawing conclusions on the association between personality and health in children, since personality is likely to change from childhood into adulthood.

This review also has some strengths. It is the first review that reports about the effects of Type D personality in the general population. Furthermore, all available literature on the subject matter was systematically reviewed and we managed to retrieve hard copies of all articles that fulfilled our selection criteria. Finally, the methodological quality of each of the selected articles was assessed with an 11-item standardised checklist of predefined criteria by both investigators.

The available evidence suggests that Type D is a vulnerability factor that not only affects people with medical conditions, but also apparently healthy individuals from the general population. Consequently, additional attention is justified for those with a Type D personality because they are at risk for work-related problems and a lower mental and physical health status. Although Type D is a stable construct [49], this does not imply that the individual's level of distress cannot be modified. Individuals with a Type D personality have a limited ability to cope adequately with stressful life events [50], and for this reason may benefit from psychological interventions that are aimed towards improving their coping skills in order to decrease the acute and chronic stress that they experience and thus to decrease their work-related problems and increase their mental and somatic health status. Future intervention trials are needed to study the extent to which interventions are able to decrease work-related problems and increase their mental and somatic health status among various people with a Type D personality.

If anything, this review suggests that Type D personality is a vulnerability factor for general psychological distress that may not only affect people with medical conditions, but also affects the health status of individuals from the general population. This review thereby provides evidence that Type D personality is not just a state of mind that people develop in reaction to the diagnosis of a medical condition, but rather represents a broad personality construct that is prevalent in a large subgroup of the general population. Consequently, it may be an important vulnerability factor to assess in future studies on work-related problems and mental and somatic health status in the general population.

## Abbreviations

(C*o*RPS): Center of Research on Psychology in Somatic diseases.

## Competing interests

The authors declare that they have no competing interests.

## Authors' contributions

The concept of this review was designed by JD. After that, both authors reviewed the available literature and checked the quality of the articles that were included in this review. FM wrote the first draft of this paper and JD supervised the writing process. Both authors approved the final version of this manuscript.

## References

[B1] Denollet J DS14: standard assessment of negative affectivity, social inhibition, and Type D personality Psychosom Med 2005 67 1 89 97 10.1097/01.psy.0000149256.81953.49 15673629

[B2] Kupper N Denollet J Type d personality as a prognostic factor in heart disease: assessment and mediating mechanisms J Pers Assess 2007 89 3 265 276 18001227 10.1080/00223890701629797

[B3] Denollet J Personality, emotional distress and coronary heart disease Eur J Pers 1997 11 343 357 10.1002/(SICI)1099-0984(199712)11:5<343::AID-PER305>3.0.CO;2-P

[B4] Denollet J Sys SU Stroobant N Rombouts H Gillebert TC Brutsaert DL Personality as independent predictor of long-term mortality in patients with coronary heart disease Lancet 1996 347 8999 417 421 10.1016/S0140-6736(96)90007-0 8618481

[B5] Denollet J Vaes J Brutsaert DL Inadequate response to treatment in coronary heart disease: adverse effects of type D personality and younger age on 5-year prognosis and quality of life Circulation 2000 102 6 630 635 10931802 10.1161/01.cir.102.6.630

[B6] Pedersen SS Lemos PA van Vooren PR Liu TK Daemen J Erdman RA Smits PC Serruys PW van Domburg RT Type D personality predicts death or myocardial infarction after bare metal stent or sirolimus-eluting stent implantation: a Rapamycin-Eluting Stent Evaluated at Rotterdam Cardiology Hospital (RESEARCH) registry substudy J Am Coll Cardiol 2004 44 5 997 1001 10.1016/j.jacc.2004.05.064 15337209

[B7] Martens EJ Mols F Burg MM Denollet J Type D personality predicts clinical events after myocardial infarction, above and beyond disease severity and depression J Clin Psychiat 2009 in press 10.4088/JCP.08m04765blu 20156412

[B8] Borkoles E Polman R Levy A Type-D personality and body image in men: The role of exercise status Body Image 2009 in press 19945926 10.1016/j.bodyim.2009.10.005

[B9] De Fruyt F Denollet J Type D personality: A Five Factor Model perspective Psychol Health 2002 17 5 671 683 10.1080/08870440290025858

[B10] de Gelder B Riet WA van de Grezes J Denollet J Decreased differential activity in the amygdala in response to fearful expressions in Type D personality Neurophysiol Clin 2008 38 3 163 169 10.1016/j.neucli.2008.03.002 18539249

[B11] Habra ME Linden W Anderson JC Weinberg J Type D personality is related to cardiovascular and neuroendocrine reactivity to acute stress J Psychosom Res 2003 55 3 235 245 10.1016/S0022-3999(02)00553-6 12932797

[B12] Hanebuth D Meinel M Fischer JE Health-related quality of life, psychosocial work conditions, and absenteeism in an industrial sample of blue- and white-collar employees: a comparison of potential predictors J Occup Environ Med 2006 48 1 28 37 10.1097/01.jom.0000195319.24750.f8 16404207

[B13] Jellesma FC Health in Young People: Social Inhibition and Negative Affect and Their Relationship with Self-Reported Somatic Complaints J Dev Behav Pediatr 2008 29 2 94 100 10.1097/DBP.0b013e31815f24e1 18285719

[B14] Kunst MJ Bogaerts S Winkel FW Peer and inmate aggression, Type D personality and post-traumatic stress among Dutch prison workers Stress and health 2009

[B15] Kupper N Denollet J de Geus EJ Boomsma DI Willemsen G Heritability of type-D personality Psychosom Med 2007 69 7 675 681 10.1097/PSY.0b013e318149f4a7 17766686

[B16] Meulenbroek LFP Thomas G Kooijman PGC de Jong FICRS Biopsychosocial impact of the voice in relation to the psychological features in female student teachers Journal of Psychosomatic Research 2010 10.1016/j.jpsychores.2009.10.002 20307705

[B17] Oginska-Bulik N Occupational stress and its consequences in healthcare professionals: the role of type D personality Int J Occup Med Environ Health 2006 19 2 113 122 10.2478/v10001-006-0016-7 17128809

[B18] Pedersen SS Yagensky A Smith OR Yagenska O Shpak V Denollet J Preliminary Evidence for the Cross-Cultural Utility of the Type D Personality Construct in the Ukraine Int J Behav Med 2009 16 2 108 15 10.1007/s12529-008-9022-4 19229633 PMC2707956

[B19] Polman R Borkoles E Nicholls AR Type D personality, stress, and symptoms of burnout: The influence of avoidance coping and social support Br J Health Psychol 2009 19930789 10.1348/135910709X479069

[B20] Preckel D von Kanel R Kudielka BM Fischer JE Overcommitment to work is associated with vital exhaustion Int Arch Occup Environ Health 2005 78 2 117 122 10.1007/s00420-004-0572-8 15726394

[B21] Smolderen KG Vingerhoets AJ Croon MA Denollet J Personality, psychological stress, and self-reported influenza symptomatology BMC Public Health 2007 7 339 10.1186/1471-2458-7-339 18036207 PMC2241613

[B22] Thomas G de Jong FI Kooijman PG Cremers CW Utility of the Type D Scale 16 and Voice Handicap Index to assist voice care in student teachers and teachers Folia Phoniatr Logop 2006 58 4 250 263 10.1159/000093182 16825778

[B23] Broek KC Van den Smolderen KG SS P Denollet J Type D personality mediates the relationship between remembered parenting and perceived health Psychosomatics 2009 in press 20484719 10.1176/appi.psy.51.3.216

[B24] Van Hiel A De Clercq B Authoritarianism is good for you: right-wing authoritarianism as a buffering factor for mental distress European Journal of personality 2009 23 33 50 10.1002/per.702

[B25] Williams L O'Carroll RE O'Connor RC Type D personality and cardiac output in response to stress Psychology and Health 2008 10.1080/08870440701885616 20205007

[B26] Williams L O'Connor RC Howard S Hughes BM Johnston DW Hay JL O'Connor DB Lewis CA Ferguson E Sheehy N Grealy MA O'Carroll RE Type-D personality mechanisms of effect: The role of health-related behavior and social support J Psychosom Res 2008 64 1 63 69 10.1016/j.jpsychores.2007.06.008 18158001

[B27] Mols F Vingerhoets AJ Coebergh JW Poll-Franse LV van de Quality of life among long-term breast cancer survivors: A systematic review European Journal of Cancer 2005 41 17 2613 2619 10.1016/j.ejca.2005.05.017 16226458

[B28] Den Oudsten BL Van Heck GL De Vries J Quality of life and related concepts in Parkinson's disease: a systematic review Mov Disord 2007 22 11 1528 1537 10.1002/mds.21567 17523198

[B29] Pedersen SS Denollet J Type D personality, cardiac events, and impaired quality of life: a review Eur J Cardiovasc Prev Rehabil 2003 10 4 241 248 10.1097/00149831-200308000-00005 14555878

[B30] Denollet J Sys SU Brutsaert DL Personality and mortality after myocardial infarction Psychosom Med 1995 57 6 582 591 8600485 10.1097/00006842-199511000-00011

[B31] Pedersen SS Denollet J Validity of the Type D personality construct in Danish post-MI patients and healthy controls J Psychosom Res 2004 57 3 265 272 10.1016/S0022-3999(03)00614-7 15507253

[B32] Aquarius AE Denollet J Hamming JF De Vries J Role of disease status and Type D personality in outcomes in patients with peripheral arterial disease Am J Cardiol 2005 96 7 996 1001 10.1016/j.amjcard.2005.05.059 16188531

[B33] Pedersen SS Denollet J Is Type D personality here to stay? Emerging evidence across cardiovascular disease patient groups Current Cardiology Reviews 2006 2 205 213 10.2174/157340306778019441

[B34] Denollet J Schiffer AA Kwaijtaal M Hooijkaas H Hendriks EH Widdershoven JW Kupper N Usefulness of Type D personality and kidney dysfunction as predictors of interpatient variability in inflammatory activation in chronic heart failure Am J Cardiol 2009 103 3 399 404 10.1016/j.amjcard.2008.09.096 19166697

[B35] Mols F Denollet J Type D personality among non-cardiovascular patient populations: A systematic review General Hospital Psychiatry 2010 32 66 72 10.1016/j.genhosppsych.2009.09.010 20114130

[B36] Barnett MD Ledoux T Garcini LM Baker J Type D Personality and chronic pain: Construct and concurrent validity of the DS14 J Clin Psychol Med Settings 2009 16 2 194 199 10.1007/s10880-009-9152-0 19266270

[B37] Simson U Nawarotzky U Porck W Friese G Schottenfeld-Naor Y Hahn S Scherbaum WA Kruse J [Depression, anxiety, quality of life and Type D pattern among inpatients suffering from diabetic foot syndrome] Psychother Psychosom Med Psychol 2008 58 2 44 50 10.1055/s-2007-971001 17828682

[B38] Spindler H Kruse C Zwisler AD Pedersen SS Increased anxiety and depression in Danish cardiac patients with a Type D personality: Cross-Validation of the Type D Scale (DS14) Int J Behav Med 2009 16 2 98 107 10.1007/s12529-009-9037-5 19322662 PMC2707957

[B39] Schiffer AA Pedersen SS Widdershoven JW Denollet J Type D personality and depressive symptoms are independent predictors of impaired health status in chronic heart failure Eur J Heart Fail 2008 10 9 922 930 10.1016/j.ejheart.2008.07.010 18942177

[B40] Schiffer AA Denollet J Widdershoven JW Hendriks EH Smith OR Failure to consult for symptoms of heart failure in patients with a Type D personality Heart 2007 93 7 814 818 10.1136/hrt.2006.102822 17344329 PMC1994460

[B41] Pelle AJ Schiffer AA Smith OR Widdershoven JW Denollet J Inadequate consultation behavior modulates the relationship between Type D personality and impaired health status in chronic heart failure Int J Cardiol 2009 in press 10.1016/j.ijcard.2008.12.086 19167768

[B42] Schiffer AA Denollet J Widdershoven JW Hendriks EH Smith OR Failure to consult for symptoms of heart failure in patients with a type-D personality Heart 2007 93 7 814 818 10.1136/hrt.2006.102822 17344329 PMC1994460

[B43] Ladwig K Emeny R Gieger C Ruf E Klopp N Illig T Meitinger T Wichmann H Single nucleotide polymorphisms associations with Type D personality in the general population: findings from the KORA K-500-Substudy APS: 2009: Psychosomatic med 2009

[B44] von Kanel R Barth J Kohls S Saner H Znoj H Saner G Schmid JP Heart rate recovery after exercise in chronic heart failure: Role of vital exhaustion and type D personality J Cardiol 2009 53 2 248 256 10.1016/j.jjcc.2008.11.008 19304130

[B45] Molloy GJ Perkins-Porras L Strike PC Steptoe A Type D personality and cortisol in survivors of acute coronary syndrome Psychosom Med 2008 70 8 863 868 10.1097/PSY.0b013e3181842e0c 18799427

[B46] Kupper N Gidron Y Winter J Denollet J Association between type D personality, depression, and oxidative stress in patients with chronic heart failure Psychosom Med 2009 71 9 973 980 10.1097/PSY.0b013e3181bee6dc 19834046

[B47] Van Craenenbroeck EM Denollet J Paelinck BP Beckers P Possemiers N Hoymans VY Vrints CJ Conraads VM Circulating CD34+/KDR+ endothelial progenitor cells are reduced in chronic heart failure patients as a function of Type D personality Clin Sci (Lond) 2009 117 4 165 172 10.1042/CS20080564 19173675

[B48] Bhattacharyya MR Perkins-Porras L Whitehead DL Steptoe A Psychological and clinical predictors of return to work after acute coronary syndrome Eur Heart J 2007 28 2 160 165 10.1093/eurheartj/ehl440 17185305

[B49] Martens EJ Kupper N Pedersen SS Aquarius AE Denollet J Type D personality is a stable taxonomy in post-MI patients over an 18-month period J Psychosom Res 2007 63 5 545 550 10.1016/j.jpsychores.2007.06.005 17980229

[B50] Doering LV Dracup K Caldwell MA Moser DK Erickson VS Fonarow G Hamilton M Is coping style linked to emotional states in heart failure patients? J Card Fail 2004 10 4 344 349 10.1016/j.cardfail.2003.10.001 15309703

